# Pharmacomicrobiomics of GLP-1 receptor agonist gastrointestinal tolerability: integrating host genetics and the gut microbiome

**DOI:** 10.3389/frmbi.2026.2016564

**Published:** 2026-10-27

**Authors:** Shreyas V. Kumbhare, Inti Pedroso, Ranjan Sinha, Daniel E. Almonacid

**Affiliations:** Digbi Health, Mountain View, CA, United States

**Keywords:** gastrointestinal tolerability, gene-by-environment interaction, GIPR, GLP-1 receptor agonist, GLP1R, gut microbiome, pharmacogenomics, pharmacomicrobiomics

## Abstract

Gastrointestinal (GI) adverse events, such as nausea, vomiting, diarrhea, and constipation, remain a dominant barrier to the sustained use of GLP-1 receptor agonists (GLP-1 RAs), driving discontinuation independently of the drug’s metabolic benefits. Two host-side biological systems have been independently established as determinants of this variability. The first is host genetics, anchored by a 2026 genome-wide association study in which GLP1R variants predicted both weight-loss efficacy and nausea or vomiting risk, while a GIPR signal for vomiting was restricted to tirzepatide users. The second system is the gut microbiome, which regulates endogenous GLP-1 secretion through short-chain fatty acid and bile acid signaling and is itself reshaped by GLP-1 RA exposure. Pharmacomicrobiomics integrates host genetic and microbiome data to explain drug response. In the literature surveyed for this review, these two determinants are consistently examined in parallel rather than jointly: we did not identify a study, in GLP-1 RA therapy or in another drug class, that fit a ‘host-genotype-by-microbiome’ interaction model against a drug tolerability outcome. Published methodological studies have suggested why such a study would be challenging: both interaction detection and the measurement unreliability associated with single-timepoint microbiome profiling impose substantial sample size penalties. This review summarizes what host genetics and the microbiome individually do and do not explain about GLP-1 RA tolerability, and describes the study design features, paired genotyping, repeat-sample metagenomics, and prospective symptom capture that a future joint analysis would require.

## 1 Introduction

GLP-1 receptor agonists (GLP-1 RAs), such as semaglutide, liraglutide, exenatide, dulaglutide, and the dual GIP/GLP-1 co-agonist tirzepatide, have transformed the pharmacological management of type 2 diabetes and obesity. Their principal limitation is not efficacy but tolerability: pooled across 101 randomized controlled trials (RCTs) (with 57,684 participants), GLP-1 RA therapy was found to produce nausea in 22.8% of patients (risk ratio 2.90 versus placebo), vomiting in 9.1% (RR 3.60), diarrhea in 13% (RR 1.97), and constipation in 7.4% (RR 2.41) (Joy et al., 2026). In the pooled STEP 1–3 trials, 4.3% of semaglutide-treated participants permanently discontinued due to a GI adverse event (Wharton et al., 2022), a modest proportion that compounds across tens of millions of users. These symptoms do not appear to be the mechanism of benefit: less than one percentage point of the additional 7.6-14.4% weight loss on semaglutide was mediated by GI adverse events (Wharton et al., 2022; Rubino et al., 2025). The physiological driver is delayed gastric emptying and reduced small-intestinal transit, mediated through peripheral vagal afferent and central area postrema pathways (Hiramoto et al., 2024), yet its magnitude correlates weakly with symptom burden. The mechanism therefore explains the class-wide tendency toward GI symptoms without explaining why any given individual tolerates the drug well or poorly (Jalleh et al., 2024).

Two host-side systems are independently implicated in this inter-individual variability. The first is host genetics. The second is the gut microbiome, which is mechanistically positioned on both sides of the relationship: it regulates endogenous GLP-1 secretion, which GLP-1 RA therapy pharmacologically amplifies, and is itself remodeled by GLP-1 RA exposure. Pharmacomicrobiomics — the study of how the gut microbiome modifies, and is modified by, drug response — has rapidly matured for drugs whose disposition is directly microbially mediated, such as digoxin and levodopa. Throughout, tolerability refers specifically to gastrointestinal adverse events and the discontinuation they drive, not to other adverse effects of this drug class. GLP-1 RAs are not microbially metabolized in that sense; instead, their pharmacomicrobiomic relevance runs through host genetic susceptibility and microbiome-mediated GI physiology acting in parallel, and potentially in interaction, on a shared tolerability outcome.

This mini review advances three claims. First, host genetics and the gut microbiome are each independently established as non-trivial predictors of GLP-1 RA GI tolerability. Second, their interaction—whether a person’s genetic risk for GI intolerance is amplified or buffered by their microbiome composition—appears not to have been examined in this drug class or in any other with a comparable evidence base. Third, this gap reflects study design and cost rather than mere neglect, since detecting a multiplicative gene-by-microbiome interaction requires severalfold more participants than detecting either main effect, and single-timepoint microbiome measurement has poor test-retest reliability. **Figure 1** summarizes the argument and the study design it implies, and **Table 1** sets out the evidence and its certainty across the domains reviewed.

**Figure 1:**
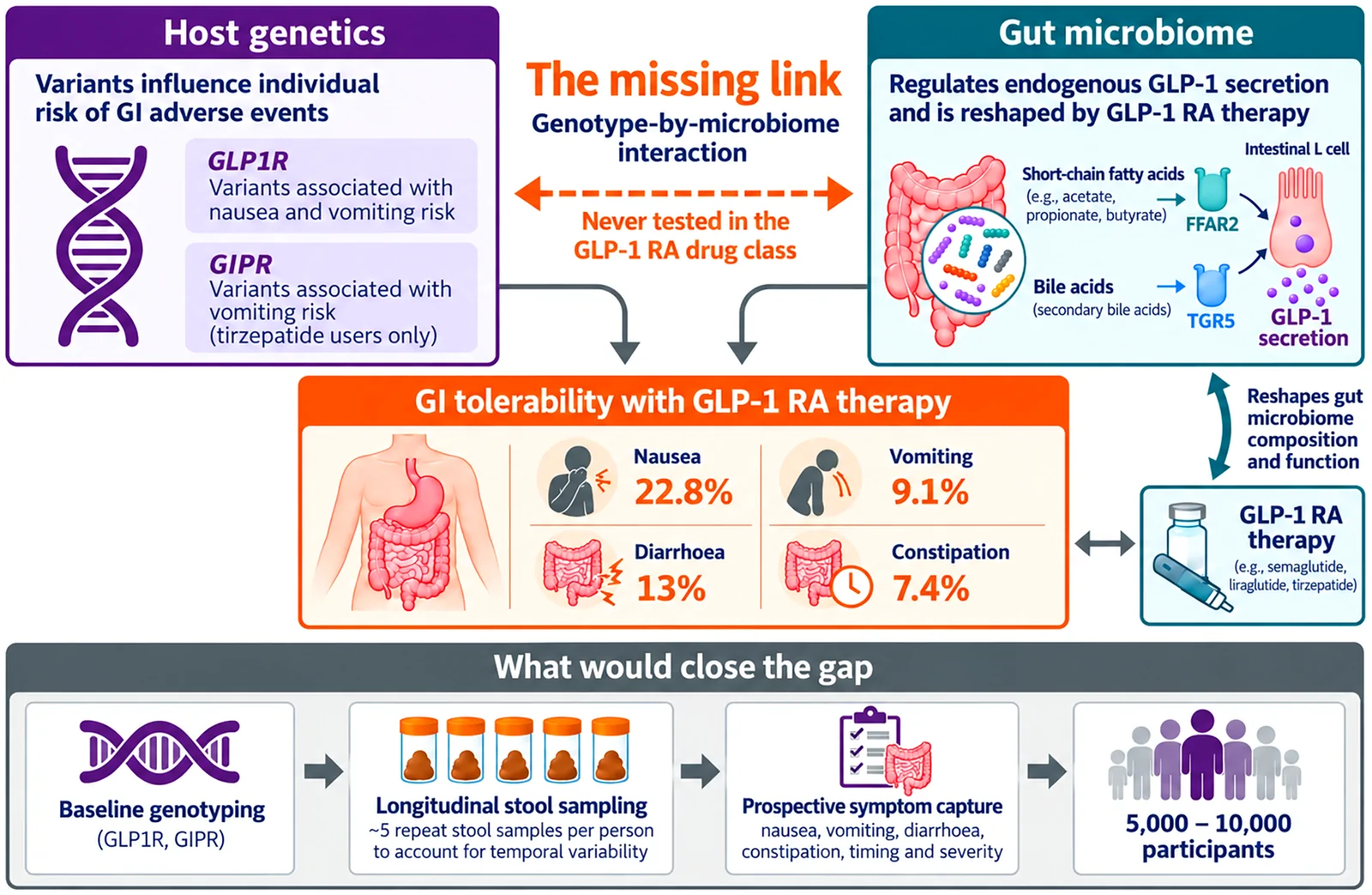
Host genetics and the gut microbiome as parallel, and potentially interacting, determinants of GLP-1 receptor agonist gastrointestinal tolerability. Host genetic variation at the GLP1R (nausea and vomiting) and GIPR (vomiting, reported only in tirzepatide users) receptors and the gut microbiome, which regulates endogenous GLP-1 secretion through short-chain fatty acid signaling via FFAR2 and bile acid signaling via TGR5 on intestinal L cells, are each independently associated with gastrointestinal tolerability. In turn, GLP-1 receptor agonist therapy reshapes microbiome composition and function, making the drug-microbiome relationship bidirectional. Pooled adverse-event frequencies are from 101 randomized controlled trials (Joy et al., 2026). The dashed arrow marks the genotype-by-microbiome interaction, which we did not identify as having been tested against a tolerability outcome in this or any other drug class. The lower panel sets out the study design features such a test would require, with the participant range derived from the illustrative arithmetic in section 4.2 rather than from a published power calculation.

**Table 1:** Summary of the key evidence and level of certainty found in the domains reviewed.

Domain	Central finding	Best evidence (design, N)	Strength of evidence	Key reference(s)	Key unanswered question
GI adverse-event burden	Nausea 22.8%, vomiting 9.1%, diarrhea 13%, constipation 7.4% (RR 2.0-3.6 vs. placebo)	Meta-analysis, 101 RCTs, N = 57,684	High	Joy et al., 2026	Which patients account for the burden? Incidence is established; the determinants of individual susceptibility are not.
GLP1R pharmacogenomics	rs10305420 predicts weight-loss efficacy; distinct index variants predict nausea and vomiting	GWAS; 27,885 surveyed, 15,237 in efficacy GWAS after QC	Moderate	Su et al., 2026	Do the nausea and vomiting variants replicate outside European-ancestry cohorts, and against record-derived rather than self-reported outcomes?
GIPR pharmacogenomics	rs71338792 predicts vomiting, restricted to tirzepatide (dual GIP/GLP-1) users only	GWAS; tirzepatide-treated subgroup	Moderate	Su et al., 2026	Is the GIPR signal specific to dual agonism, and does it operate through the GIP-mediated attenuation of emesis seen preclinically?
Broader candidate genes (HTR3A/B, GPBAR1, SCN5A)	No candidate-gene study identified in GLP-1 RA-treated cohorts; the one genome-wide scan reported no signal at any of them	Targeted narrative search (no study identified) + GWAS	Low	This review; Su et al., 2026	Do these loci carry any signal in a GLP-1 RA-treated cohort with prospectively captured GI outcomes? None of these has been genotyped against this outcome.
Microbiome regulation of GLP-1 secretion	SCFA and bile acid signaling raise GLP-1 secretion; mechanism is receptor-specific, not a general property	RCT (inulin-propionate ester), N = 60	Moderate	Chambers et al., 2015	Does raising endogenous GLP-1 secretion change tolerability, or only the hormonal readout? No study has linked the secretion axis to a symptom endpoint.
GLP-1 RA-induced microbiome remodeling	The best-designed RCT found no detectable diversity change; evidence base overall animal-dominated and metformin-confounded	RCT, N = 51	Moderate	Smits et al., 2021	Does any compositional change precede, follow, or merely accompany symptoms, once weight loss and concomitant metformin are separated?
Host genetics → microbiome composition (general population)	Eight loci with study-wide significant associations (confirming LCT, ABO, FUT2; new: MUC12, CORO7-HMOX2, SLC5A11, FOXP1, FUT3-FUT6); 11 of the 15 associations were replicated at six loci. Heritability 9% (Shannon index) and 20% (richness)	GWAS, N = 16,017 discovery + 12,652 replication	Moderate	Dekkers et al., 2026	Do the loci that shape microbiome composition in general populations behave in the same way under drug exposure?
Genotype × microbiome interaction on drug tolerability	No study identified, in any drug class, fitting this interaction model against a tolerability outcome	Targeted narrative search (no study identified)	Very low (evidence gap)	This review	Does genetic risk for GI intolerance depend on microbiome composition? Untested in this or any other drug class.
Feasibility of closing the gap	Illustrative scale: ~5,000-10,000 participants with repeat-sample metagenomics	Methodological synthesis (not a published power calculation)	N/A (derived)	Smith and Day, 1984; Wong et al., 2003; Vandeputte et al., 2021	What is the true required sample size under a realistic design, rather than the illustrative arithmetic offered here?
Clinical translation	No genetics- or microbiome-guided program identified as reporting a GLP-1 RA tolerability outcome	Targeted narrative search (no study identified)	Very low (evidence gap)	This review	Do any genetics- or microbiome-guided interventions change prespecified tolerability endpoints?

## 2 Host genetic determinants of GLP-1 RA gastrointestinal tolerability

### 2.1 GLP1R and GIPR: the two genome-wide significant loci

The genetic architecture of GLP-1 RA tolerability was first mapped on a genome-wide scale in 2026, using self-reported side effect and outcome data from 27,885 GLP-1 RA users in the 23andMe research cohort, of whom 15,237 formed the efficacy GWAS sample after quality control (Su et al., 2026). Two loci reached genome-wide significance. At GLP1R, the missense variant rs10305420 (p.Pro7Leu) was associated with greater weight-loss efficacy (P = 2.9 × 10^-^¹^0^), while GLP1R index variants distinct from the efficacy signal were associated with medication-related nausea (rs9357296, OR 1.36) and vomiting (rs11760106, OR 1.57). The second locus was GIPR (rs71338792, OR 1.84 for vomiting), reported as restricted to tirzepatide users; the same study found no semaglutide-versus-tirzepatide difference for the GLP1R side effect variants, indicating that the drug-specific pattern is confined to GIPR. GI intolerance genetics for incretin therapy may therefore be partly receptor-target specific rather than uniformly class-wide.

Two features temper the extrapolation of these effect sizes. A combined genetic and non-genetic model explained only approximately 25% of weight-loss efficacy in held-out test sets, with sex, drug type, dose, and treatment duration accounting for more variance than any genetic variant. The discovery cohort was 78.3% European ancestry and predominantly female, and self-reported weight change (−11.8%) was approximately double the record-derived estimate (−5.8%). These effect sizes should not be treated as established outside European-ancestry populations, and this skew is the single largest constraint on generalizing the genetic evidence reviewed here.

Independent replication of GLP1R candidate variants has been mixed. In a 4,571-participant pharmacogenomic GWAS of glycemic response, rs6923761 showed a modest per-allele HbA1c difference, while rs10305420 showed none, and an ARRB1 missense variant was independently associated with greater HbA1c reduction, implicating downstream signaling rather than GLP1R sequence alone (Dawed et al., 2023). In two similarly designed Chinese cohorts, rs3765467 was associated with GI adverse reactions in one cohort (Long et al., 2022) but not in the other (Guan et al., 2022). rs6923761 is directionally inconsistent within a single trial: carriers lost more weight on liraglutide, yet they showed decreased incretin responsiveness (Mashayekhi et al., 2025). Additionally, rs6923761 independently predicts baseline obesity risk (Michałowska et al., 2022), so associations using percentage weight change may partly reflect confounding by adiposity. The rare loss-of-function variant rs367543060 proved absent in the one GLP-1 RA-treated cohort genotyped for it (Kyriakidou et al., 2024): examined, not untested.

### 2.2 Candidate-gene evidence beyond GLP1R

Beyond GLP1R and GIPR, the candidate genes most often proposed on mechanistic grounds remain unexamined in this drug class. We did not identify a candidate-gene study testing the serotonin-receptor genes HTR3A/HTR3B, the bile acid receptor gene GPBAR1/TGR5, or the motility gene SCN5A in a GLP-1 RA-treated cohort with GI side effect outcomes, and the one genome-wide scan available reported no signal at any of these.

Each gene has supporting evidence from other emetic or GI motility contexts, none of it tested in incretin therapy. HTR3A and HTR3B variants predict chemotherapy-induced and postoperative nausea and vomiting (Yeo et al., 2025; Kim et al., 2015). However, Clinical Pharmacogenetics Implementation Consortium (CPIC) declines to recommend HTR3 genotyping, calling the evidence insufficient even in its home domain (Bell et al., 2017). The GPBAR1/TGR5 genotype is associated with colonic transit and fecal bile acid excretion in IBS (Camilleri et al., 2014), and TGR5 activation stimulates GLP-1 secretion (Thomas et al., 2009), a link not examined end-to-end in treated patients. Rare SCN5A loss-of-function variants are enriched in patients with IBS (Beyder et al., 2014; Strege et al., 2018); however, an IBS GWAS of 53,400 cases found no genome-wide significant signal at SCN5A, GPBAR1, or any serotonin-receptor locus (Eijsbouts et al., 2021). Motion sickness offers a calibration: a GWAS of 80,494 individuals identified 35 significant SNPs whose propensity score explained 2.9% of the variance (Hromatka et al., 2015).

## 3 The gut microbiome as a bidirectional modulator of GLP-1 biology

### 3.1 Microbiome regulation of endogenous GLP-1 secretion

The gut microbiome regulates GLP-1 secretion through convergent mechanisms. Short-chain fatty acids (SCFAs) from microbial fermentation of dietary fiber stimulate L-cell GLP-1 release, and SCFA-triggered secretion is impaired in Ffar2/Ffar3-knockout mice (Tolhurst et al., 2012; Psichas et al., 2015). The mechanism is not simply receptor agonism: in the perfused rat colon, SCFA-evoked secretion was not reproduced using FFAR2/3-selective agonists, and it was abolished by metabolic inhibitors, suggesting that SCFAs act as colonocyte energy substrates (Christiansen et al., 2018). In humans, a 24-week RCT of inulin-propionate ester increased postprandial GLP-1 and peptide YY (PYY) levels and improved insulin sensitivity (Chambers et al., 2015), whereas a galacto-oligosaccharide RCT achieved a fivefold increase in Bifidobacterium levels with no change in GLP-1 (Canfora et al., 2017): a microbiota shift does not guarantee a hormonal one.

Bile acids constitute a second, mechanistically distinct axis, acting through GPBAR1/TGR5. Intrarectal infusion of bile acids increased plasma GLP-1 sevenfold in a human crossover study (Adrian et al., 2012), and a 28-day RCT of colon-targeted conjugated bile acids increased postprandial GLP-1 and C-peptide (Calderon et al., 2020). Signaling through the nuclear receptor FXR runs counter, decreasing GLP-1 secretion *in vitro* (Trabelsi et al., 2015); that bile acids increase GLP-1 is therefore receptor-specific, not a general property of the bile acid pool. A large dietary intervention study linked high-fiber intake and SCFA-producing enrichment to elevated GLP-1, with gnotobiotic mouse transplantation experiments validating the chain (Zhao et al., 2018), and a Mendelian randomization study supports a causal effect of microbial butyrate production on the post-glucose insulin response (Sanna et al., 2019).

The relationship is not monotonic: germ-free mice have higher plasma GLP-1 levels and slower small-intestinal transit times than conventionally raised mice (Wichmann et al., 2013); in addition, colonization downregulates ileal L-cell GLP-1 content (Arora et al., 2018).

### 3.2 GLP-1 RA-induced remodeling of the gut microbiome

The reverse direction rests on a smaller, more heterogeneous evidence base. The best-designed human study, a 12-week, double-blind, placebo-controlled RCT of liraglutide (N = 51), found no change in alpha diversity or beta diversity despite clear glycemic and bile acid effects (Smits et al., 2021). A 2025 systematic review found that five of eight human liraglutide studies reported no compositional alterations. The base is animal-dominated: of 38 studies, only 9 were in humans, with no human semaglutide study identified (Gofron et al., 2025), which a dated search at revision confirmed (**Supplementary Table S2**). We did not identify a published human study of tirzepatide’s effect on microbiota composition; the findings located here are rodent-derived (Cadena-Ullauri et al., 2026).

Positive findings are frequently confounded by weight loss or the concomitant use of metformin. The first human report found increased Akkermansia with liraglutide relative to metformin (Wang et al., 2018); however, a comparable mouse study found that the increase tracked the degree of weight loss, with no weight-matched control group (Madsen et al., 2019). In the MIGHTY trial, the liraglutide arm carried metformin throughout, and the authors attribute the taxa shifts to both regimens (Glaros et al., 2025). Metformin itself is a causally active, microbiome-altering drug (Wu et al., 2017) that produced a larger Faecalibacterium increase than liraglutide in a head-to-head comparison (Ying et al., 2023). Even a robust shift need not be meaningful: Akkermansia muciniphila rises after bariatric surgery without metabolic improvement (Dao et al., 2019). Most relevant here, this literature is oriented toward efficacy rather than tolerability: we did not identify a human study that tested whether baseline or drug-induced composition predicts GI adverse events, and a 2026 narrative review similarly reported response-prediction studies without tolerability studies (De La Motte et al., 2026). Whether such compositional changes are causes, consequences, or correlates of tolerability is unresolved by any existing design. Because therapy itself remodels the microbiome, post-treatment composition cannot be read as a baseline predictor; separating these three requires pre-treatment sampling, a monotherapy arm, and a weight-matched comparison (**Supplementary Table S4**).

### 3.3 From microbial activity to gastrointestinal symptoms

The preceding section establishes that the microbiome regulates GLP-1 secretion, but tolerability is a symptom phenotype, and the two are distinct claims. Several microbial activities bear on symptoms directly. Fermentation generates luminal gas: prebiotic oligofructose increased breath hydrogen alongside incretin secretion and satiety in healthy adults (Cani et al., 2009), with gas volume, distension, and transit being proximate determinants of bloating and pain. Bacterial bile salt hydrolase activity sets the composition of the colonic bile acid pool (Joyce et al., 2014), and bile acid excess is itself a diarrheal mechanism responsive to GLP-1 RA therapy, although it has only been reported in a single case (Kårhus et al., 2022). Metabolites beyond SCFAs and bile acids also reach relevant receptors: the bacterial tryptophan metabolite indole modulates incretin secretion from L cells (Chimerel et al., 2014).

The efferent arm is equally plausible. GLP-1 receptor signaling on vagal afferents governs food intake and glycemia (Krieger et al., 2016). The anorectic response to exogenous GLP-1 is lost after truncal vagotomy (Plamboeck et al., 2013), and hindbrain GLP1R circuits for satiety and aversion are anatomically dissociable (Huang et al., 2024). Consistent with this dissociation, GIP receptor agonism attenuated GLP-1 RA-induced nausea and emesis in preclinical models while preserving reduced intake (Borner et al., 2021) — a candidate mechanism for the tirzepatide-restricted GIPR signal discussed in §2.1.

It is established that microbiome composition can predict drug-induced GI adverse events in other drug classes, if not this one: bacterial β-glucuronidase reactivates the irinotecan metabolite SN-38 in the gut lumen, and composition is associated with chemotherapy-induced mucositis and checkpoint-inhibitor colitis (**Supplementary Table S3**). In principle, the phenotype is microbially tractable; however, it has not been tested for this drug class.

## 4 The genetics and microbiome intersection: an unexamined interaction

### 4.1 Precedent across drug classes

If host genetics and the gut microbiome are each independently implicated in GLP-1 RA tolerability, the natural next question is whether they interact, that is, whether a person’s genetic risk of GI intolerance is amplified or buffered by their microbiome composition. We surveyed this across drug classes for which a joint genetic and microbial determinant of drug response has been proposed, including metformin-induced GI intolerance, levodopa pharmacokinetics, digoxin inactivation, and chemotherapy-induced mucositis. Of the literature located for this review, we did not find any study fitting a multiplicative host-genotype-by-microbiome interaction model against a drug tolerability or adverse-event outcome.

Three studies approach this design. The only human study to fit an explicit genotype-by-microbiome interaction term analyzed intestinal mucosal gene expression rather than drug response (36 rectal and 30 ileal interactions among 86 individuals; Nirmalan et al., 2026, preprint); the model is fittable in humans, but the outcome is wrong. The closest attempt to investigate GI intolerance to a drug used blood mRNA rather than germline genotype in 18 samples and did not fit an interaction term (Zhang et al., 2023). Microbial pathways determine digoxin inactivation (Haiser et al., 2013) and levodopa metabolism (Maini Rekdal et al., 2019); however, neither tested for a host genetic modifier.

Available datasets are equally limiting. The largest genotype-plus-microbiome resources—MiBioGen (18,340; Kurilshikov et al., 2021), a Swedish-Norwegian GWAS (16,017 plus 12,652; Dekkers et al., 2026), the Dutch Microbiome Project (7,738; Lopera-Maya et al., 2022), and FINRISK (5,959; Qin et al., 2022)—are general-population cohorts without a GLP-1 RA exposure arm, and none pairs host genotyping, repeat-sample metagenomics, and prospective symptom capture in treated patients. A 2026 clinical pharmacology review independently concluded that discrepancies across studies likely reflect unaccounted host-genetic and microbiome variation, and called for the two to be modeled jointly (Kamath et al., 2026).

### 4.2 Methodological constraints on a joint analysis

Three methodological findings bear on the scale that such an analysis would require. First, detecting a multiplicative interaction requires at least four times the sample size of a study powered for a main effect of the same magnitude (Smith and Day, 1984); this rule was derived for case-control designs with a dichotomous exposure, so its transfer to a continuous microbiome feature is an approximation. Second, measurement error in the interacting exposure inflates the requirement sharply: under Wong’s illustrative parameters, detecting the interaction requires 150,989 participants when exposure and outcome reliability coefficients are 0.3 and 0.4, fewer than 10,000 participants at 0.7/0.7, and below 5,000 participants with repeated measurement (Wong et al., 2003). Third, gut microbiome features sit squarely in this low-reliability regime: daily profiling of 713 stool samples from 20 women found that, for 78% of genera, day-to-day abundance varies more within individuals than between them. The study also identified the optimal accuracy-effort trade-off to be approximately five samples per person (Vandeputte et al., 2021).

Read together, these findings suggest an order of magnitude rather than a requirement: single-timepoint taxon-level testing would call for 10^4^–10^5^ treated participants, while five repeat samples with pathway-level features would lower this to 5,000–10,000. This is an arithmetic combination of three independently sourced parameters, illustrative only; it is not a power calculation and should not be read as one.

### 4.3 An empirical test of one link in the proposed pathway

One finding tests a specific link in this pathway. OR51E1-OR51E2, which encodes short-chain fatty acid chemosensors in enteroendocrine cells, was associated with microbial richness in the largest microbiome GWAS, but showed no association with fasting or post-glucose plasma GLP-1 in up to 3,514 participants (Dekkers et al., 2026). The authors call this a “somewhat limited sample”, so the null is uninformative rather than providing evidence against the link, and it tests one candidate link in a drug-naive population, rather than an interaction on tolerability.

## 5 Scope and limitations of this review

Genetics and the microbiome are not the only, and may not be the dominant, determinants of who tolerates GLP-1 RA therapy: in the largest efficacy GWAS, genetic variants explained approximately 25% of the variance in weight-loss response after accounting for sex, dose, drug type and duration (Su et al., 2026). The titration protocol appears to be a comparably large lever: a randomized open-label trial found that a 16-week flexible semaglutide escalation reduced withdrawal driven by GI adverse events to 2%, versus 19% on the standard 8-week schedule, with equivalent final HbA1c (Eldor et al., 2025), a protocol-driven effect larger than any between-drug or between-genotype comparison in this literature, and the largest modifiable determinant of tolerability identified in this review. Behavioral, dietary, and psychological factors are not addressed by either data type.

This is a narrative review, with the literature identified through a targeted search of multiple databases current up to August 2026 rather than through a preregistered systematic protocol with archived search strings. Where we report not having identified a study of a given design, that statement describes the corpus retrieved for this review and is not an assertion that no such study exists. The synthesis method — the statement graph, its certainty distribution, and the dated searches supporting each claim of absence — is set out in Supplementary Methods and **Supplementary Tables S1**-**S3**. Notably, no statement in either of the two streams closest to this review’s question reached High certainty.

## 6 Clinical and translational implications

Management strategies for GLP-1 RA GI intolerance are almost entirely untested by randomized comparison. The dominant consensus recommends slower escalation, temporary dose reduction, small low-fat meals, and domperidone over metoclopramide in older patients; this was a Novo Nordisk-funded consensus that had no randomized support when issued (Gorgojo-Martínez et al., 2022), and the flexible titration RCT mentioned above is the first randomized evidence consistent with its core recommendation. Antiemetic prophylaxis is similarly underpowered: the only published report of mirtazapine for GLP-1 RA intolerance is a single case study (Khan et al., 2025), and while mirtazapine improves an analogous symptom cluster in functional dyspepsia (Tack et al., 2016), it reliably causes weight gain (Serretti and Mandelli, 2010), which is pharmacodynamic antagonism to the therapeutic goal.

The closest evaluation of genetics- and microbiome-informed digital care is a retrospective, single-arm, author-affiliated study of 177 participants, with no control arm, no randomization, and no GLP-1 RA users (Kumbhare et al., 2022). We identified no peer-reviewed study of any genetics- or microbiome-guided program reporting GLP-1 RA tolerability, nausea incidence, dose-escalation success, or persistence as prespecified outcomes. Therefore, the rationale for precision tolerability management rests on mechanistic plausibility rather than clinical outcome data, and is best presented to patients and prescribers on that basis.

Three questions follow for translation. First, what endpoint should an early-phase trial use? Dose-escalation success, prospectively captured symptom severity, and persistence at 3 to 6 months are practical candidates, each measurable before weight outcomes mature. Second, should genotyping or microbiome profiling be offered now to manage tolerability? On the evidence surveyed, no: no test has been shown to alter a tolerability outcome, and the titration effect above is both larger and already actionable. Third, cost-effectiveness is unaddressed — we identified no analysis costing genotyping or repeat metagenomics against the simpler comparator of slower escalation.

## 7 Conclusion and future directions

Host genetics and the gut microbiome are each independently implicated in GLP-1 RA gastrointestinal tolerability: GLP1R and GIPR are the genome-wide significant loci reported to date, with the GIPR signal restricted to dual GIP/GLP-1 agonism, while the microbiome regulates endogenous GLP-1 secretion and is itself remodeled by therapy. Their interaction remains unexamined, here and in the other drug classes reviewed. Two features of the evidence base account for this: the genotype-plus-microbiome cohorts identified do not include GLP-1 RA exposure, and published methodological work implies interaction sample sizes exceeding those available in those cohorts.

Closing this gap requires a specific, nameable study: the prospective enrollment of GLP-1 RA initiators with baseline genotyping (at least GLP1R and GIPR), repeat-timepoint stool metagenomics at approximately five samples per person, and structured prospective symptom capture, powered for interaction detection rather than main effects and, based on our arithmetic, several thousand participants using pathway-level features (**Supplementary Table S4**). Existing biobanks should meanwhile be evaluated for retrofittable GLP-1 RA exposure through linked pharmacy and health record data, and the tirzepatide-specific GIPR signal warrants replication given its relevance to next-generation incretin co-agonists. Until such work is reported, descriptions of genetics- and microbiome-guided GLP-1 RA tolerability management rest on extrapolation from the mechanism rather than on demonstrated clinical benefit. .
