# Supplementary material for: Pharmacomicrobiomics of GLP-1 receptor agonist gastrointestinal tolerability: integrating host genetics and the gut microbiome
Source: Front Microbiomes. 2026 Oct 27;5:2016564. doi: 10.3389/frmbi.2026.2016564 (PMC13749394; doi:10.3389/frmbi.2026.2016564)
Supplement: Supplementary file 1 [file SupplementaryFile1.docx]

**Supplementary Material**

*Pharmacomicrobiomics of GLP-1 Receptor Agonist Gastrointestinal Tolerability: Integrating Host Genetics and the Gut Microbiome*

Kumbhare SV, Pedroso I, Sinha R, Almonacid DE

**Supplementary Methods: evidence synthesis by statement graph**

**1. Approach**

Evidence for this review was synthesised using a statement-graph method, a general procedure previously applied by our group to another narrative review. Every claim eligible to enter the manuscript was first extracted as a discrete statement carrying its own primary source and certainty rating, and relationships between statements were then recorded explicitly as typed, individually justified edges. The procedure suits a review whose principal contribution is negative, because it makes each claim auditable to a single source and forces disagreement between sources to be recorded rather than resolved silently in prose.

**2. Research streams**

The literature was partitioned into eight parallel research streams, each with a defined scope (Supplementary Table S1). Extraction proceeded stream by stream; relationships between statements were specified afterwards, and are predominantly within streams rather than across them. The primary extraction was compiled on 4 August 2026 and the literature is current to August 2026, except where updated at revision.

**3. Statement extraction, evidence typing and GRADE assignment**

Extraction produced 235 statements. Each carries a primary source (first author, year and, where one exists, a DOI), a free-text evidence-type label recording the study design in specific terms (for example "meta-analysis of RCTs", "mechanistic/animal", "cross-sectional (interaction eQTL; preprint)"), and a GRADE certainty rating drawn from the four standard levels. Grading is anchored to the evidence type: an automated check flags any statement whose grade is inconsistent with its design label, such as animal, mechanistic, case-report or press-release evidence graded High or Moderate, or meta-analytic or Mendelian-randomisation evidence graded Low or Very low. The resulting distribution was High 27, Moderate 77, Low 81 and Very low 50.

Statements sourced from material that has no journal article behind it — product prescribing information, sponsor trial announcements, a corporate press release, and the statements recording negative search findings — carry no DOI. Eight statements fall into this category, and a dedicated check flags any statement missing a DOI without such a justification. Where a reported quantity could not be verified from a retrievable version of record it was omitted rather than estimated.

The distribution across streams is itself informative and is reported in full in Table S1. Certainty concentrates in the stream describing gastrointestinal adverse-event epidemiology, where 19 of 42 statements are rated High, and collapses in the two streams closest to this review’s question: no statement in the drug-to-microbiome stream or in the genetics-by-microbiome intersection stream reached High certainty, and 11 of 30 statements in the former are rated Very low. The evidence is strongest where the clinical problem is described and weakest where its proposed determinants are tested.

Evidence originating from the authors’ own affiliation is tagged as such in the evidence-type field, and a dedicated check tests that tagging for completeness, so that author-affiliated sources cannot enter the synthesis unmarked.

**4. Edge construction**

55 relationships between statements were specified, each with a typed relation and a written rationale: bounds (18), contradicts (15), supports (15), extends (5) and gap (2). The bounds relation carries much of the analytical weight. It records where one finding constrains how much weight another can bear — for example where two endpoints in the same trial diverge (near-identical symptom rates but a fourfold difference in discontinuation), where a result is conditional on measurement modality (a delay seen by scintigraphy but not by an absorption test), or where a signal attenuates in a subgroup. This relation, rather than simple agreement or disagreement, is what the evidence in this field most often required.

**5. Adversarial quality assurance**

The graph was subjected to two manual adversarial review rounds, in which every edge was read specifically for error rather than for confirmation. Those rounds identified ten errors in the edge set, and further errors were corrected in the statement table, giving twelve substantive corrections in total. The error patterns found manually were then encoded as automated checks and implemented as two linting scripts, so that subsequent revisions start from a mechanically clean baseline.

The edge linter applies seven checks: structural integrity; edges touching statements already marked for exclusion; same-source pseudo-corroboration, where one primary study appears on both ends of an edge as though it were two agreeing studies; valence inversion between a relation label and its own written rationale; unhedged inference from animal to human evidence; entity mismatch, where an identifier named in a rationale appears in neither statement; and relation-type consistency for within-study contrasts. The statement linter applies nine: identifier and stream consistency, sequential identifier integrity, GRADE value validity, the grade-versus-design consistency check described above, missing DOI without justification, author-affiliation tagging completeness, cross-reference integrity, truncated text detection, and near-duplicate detection among statements sharing a DOI. Five of the edge checks are treated as decisive, and any flag they raise is corrected; the remainder are heuristic tripwires whose flags were resolved by human reading. Two false positives were examined, documented as such and deliberately retained rather than suppressed.

**6. Exclusion decisions**

Statements could be marked for exclusion from the manuscript while remaining in the graph, so that the decision stayed visible and auditable. One statement was excluded on this basis: a claim of threefold better weight loss and fivefold better tolerability under genetics- and microbiome-guided care, which originated in uncited corporate material and was inconsistent with the discontinuation rates derived from randomised trials elsewhere in the graph. Because that claim concerned the commercial context of the authors’ own affiliation, its exclusion is recorded here explicitly. Non-peer-reviewed preprints were retained only where labelled as preprints at the point of citation, and one further preprint was excluded from citation entirely.

**7. Verification searches for the absence claims**

The primary extraction was carried out under conditions in which several bibliographic interfaces were intermittently unavailable, and it therefore did not yield structured database queries with recorded hit counts. Because the review’s principal claims are negative, those specific claims were re-tested at revision using reproducible queries whose exact strings, databases, dates and yields are reported in Supplementary Table S2. Searches were run on 8 October 2026 in PubMed and in Europe PMC; Europe PMC was included because it indexes medRxiv and bioRxiv, allowing the absence claims to be tested against preprints as well as peer-reviewed literature. Every record returned was screened by title and, where the title was not decisive, by abstract.

No qualifying study was found for any absence claim. A search for the phrase forms under which a host-genotype-by-microbiome interaction would be named returned one qualifying human record, the intestinal mucosal gene-expression study already cited in the main text, whose outcome is gene expression rather than drug response; the remaining records were agricultural, animal or oncological network analyses. Restricting the same search to preprint sources returned no qualifying record, so the claim holds for preprints as well as for the peer-reviewed literature.

The same question was then put to the other drug classes in which a joint genetic and microbial determinant of drug response has been proposed (Supplementary Table S3). In each class the two determinants have been studied separately, often at considerable scale, and in none of them were they modelled jointly against a tolerability outcome. The pattern is clearest for irinotecan, where host pharmacogenetics and a microbial mechanism are each established in large independent literatures that have almost never met.

**8. Limitations of the search**

This is a narrative synthesis, not a systematic review. The statement graph makes the evidence that was used fully traceable, but it does not certify that the search which produced it was exhaustive, and no PRISMA flow is claimed. The verification searches in Tables S2 and S3 are reproducible and dated, but they were designed to test specific negative claims rather than to enumerate the field, and single-database hit counts are sensitive to query formulation. Coverage of older and smaller candidate-gene literature is acknowledged to be incomplete. Where the main text reports that no study of a given design was identified, that statement describes the corpus retrieved by these procedures; it is not an assertion that no such study exists.

**Supplementary Table S1. Research streams, statement counts and GRADE certainty distribution.**

| **Stream** | **Scope** | **Statements** | **High** | **Moderate** | **Low** | **Very low** |
| --- | --- | --- | --- | --- | --- | --- |
| S1 | GI adverse-event epidemiology and between-drug differences | 42 | 19 | 14 | 9 | 0 |
| S2 | Mechanisms of GLP-1 RA-induced GI toxicity | 30 | 2 | 12 | 6 | 10 |
| S3 | GLP1R pharmacogenomics | 26 | 0 | 11 | 12 | 3 |
| S4 | Genetic architecture beyond GLP1R | 25 | 0 | 8 | 15 | 2 |
| S5 | Microbiome to GLP-1 secretion axis | 30 | 0 | 9 | 14 | 7 |
| S6 | GLP-1 RA to microbiome composition axis | 30 | 0 | 5 | 14 | 11 |
| S7 | Genetics x microbiome intersection | 23 | 0 | 13 | 4 | 6 |
| S8 | Clinical and therapeutic implications | 29 | 6 | 5 | 7 | 11 |
| Total |  | 235 | 27 | 77 | 81 | 50 |

One statement and its edge were added during a later content-level review pass and are included in the counts above.

**Supplementary Table S2. Verification searches for the absence claims made in the main text.**

Searches were executed on the date shown and are reproducible by pasting the query string into the named database. "Records" is the hit count returned; "Qualifying" is the number meeting the design criteria of the claim after title screening and, where the title was not decisive, abstract screening. PubMed field tags: [tiab] restricts matching to title and abstract. The Europe PMC parameter SRC:"PPR" restricts results to preprint sources, which include medRxiv and bioRxiv.

| **#** | **Claim tested (main text)** | **Database** | **Query string as executed** | **Date** | **Records** | **Qualifying** | **Screening outcome** |
| --- | --- | --- | --- | --- | --- | --- | --- |
| 1 | No candidate-gene study of HTR3A/HTR3B, GPBAR1/TGR5 or SCN5A in a GLP-1 RA-treated cohort with GI side-effect outcomes (§2.2) | PubMed | ("GLP-1 receptor agonist"[tiab] OR semaglutide[tiab] OR liraglutide[tiab] OR exenatide[tiab] OR dulaglutide[tiab] OR tirzepatide[tiab]) AND (HTR3A[tiab] OR HTR3B[tiab] OR GPBAR1[tiab] OR TGR5[tiab] OR SCN5A[tiab]) | 8 Oct 2026 | 9 | 0 | All records were mechanistic, animal or narrative-review reports. None was a genotype–phenotype association study in drug-treated patients with GI outcomes. |
| 2 | No study fitting a host-genotype-by-microbiome interaction model against a drug tolerability or adverse-event outcome, in any drug class (§4.1) | PubMed | (microbiome[tiab] OR microbiota[tiab] OR metagenom*[tiab]) AND (genotype[tiab] OR genomic[tiab] OR "polygenic"[tiab] OR SNP[tiab] OR GWAS[tiab]) AND (interaction[tiab] OR "gene-by-environment"[tiab] OR "gene x environment"[tiab]) AND (drug[tiab] OR medication[tiab] OR therapy[tiab] OR treatment[tiab]) AND (tolerability[tiab] OR "adverse event*"[tiab] OR "adverse drug reaction*"[tiab] OR "side effect*"[tiab] OR intolerance[tiab]) | 8 Oct 2026 | 6 | 0 | No record fitted an interaction model against a tolerability outcome. Records concerned tumour microenvironment, phage therapy, vaccine immunogenicity, gluten intolerance and conference proceedings. |
| 3 | As claim 2, tested against preprint as well as peer-reviewed sources | Europe PMC | ((microbiome OR microbiota) AND (genotype OR "host genetic") AND interaction AND (tolerability OR "adverse event" OR "side effect")) AND (SRC:"PPR") | 8 Oct 2026 | 5 | 0 | Preprint sources only. Records concerned COVID-19 vaccine reactogenicity, a CDKL5 mouse model, MAIT cells in lung injury and COVID-19 sex differences. The absence claim therefore holds for preprints. |
| 4 | As claim 2, tested by the phrase forms under which such an interaction would be named | Europe PMC | ("gene-by-microbiome" OR "genotype-by-microbiome" OR "genotype x microbiome" OR "gene x microbiome" OR "host genotype by microbiome") | 8 Oct 2026 | 863 | 1 | The one qualifying human record is the intestinal mucosal gene-expression study already cited in the main text; its outcome is gene expression, not drug response. Remaining records were plant, rhizosphere, rumen, food-fermentation or oncology-network studies. |
| 5 | No human study testing whether baseline or drug-induced microbiota composition predicts GI adverse events on GLP-1 RA therapy (§3.2) | PubMed | ("GLP-1 receptor agonist"[tiab] OR semaglutide[tiab] OR liraglutide[tiab] OR exenatide[tiab] OR dulaglutide[tiab] OR tirzepatide[tiab]) AND (microbiome[tiab] OR microbiota[tiab]) AND (nausea[tiab] OR vomiting[tiab] OR diarrh*[tiab] OR constipation[tiab] OR tolerability[tiab] OR "adverse event*"[tiab]) | 8 Oct 2026 | 9 | 0 | Records addressed efficacy-response prediction, bile-acid diarrhoea as a treatment indication, nutrition reviews and scoping reviews. None used a GI tolerability endpoint as the outcome predicted from microbiota. |
| 6 | No published human study of tirzepatide’s effect on gut microbiota composition (§3.2) | PubMed | tirzepatide[tiab] AND (microbiome[tiab] OR microbiota[tiab]) | 8 Oct 2026 | 27 | 0 | Primary studies retrieved were rodent: diabetic kidney disease, bone loss, high-fat-diet obesity and hepatic steatosis. Remaining records were narrative reviews. No human primary study was identified. |
| 7 | No prospective genotype-guided dose-titration trial in GLP-1 RA therapy (§6) | PubMed | ("GLP-1 receptor agonist"[tiab] OR semaglutide[tiab] OR liraglutide[tiab] OR tirzepatide[tiab]) AND (genotype[tiab] OR pharmacogenom*[tiab] OR "genetic test*"[tiab]) AND (titration[tiab] OR "dose escalation"[tiab] OR guided[tiab]) | 8 Oct 2026 | 4 | 0 | Records were narrative reviews of precision pharmacotherapy and two pharmacogenomic association studies. None allocated titration by genotype. |

Interpretation. These searches were designed to falsify the review’s negative claims rather than to enumerate the field. Each returned a small, fully screenable record set, and none returned a study meeting the stated design criteria. The searches therefore confirm the claims as written; they do not establish that the underlying literature search was exhaustive, and the main text is worded accordingly.

**Supplementary Table S3. Drug classes in which a joint host-genetic and microbial determinant of drug response has been proposed, and whether an interaction between the two has been modelled.**

Record counts are PubMed hit counts for the query shown, executed on 8 October 2026. The final column records whether any retrieved study fitted a statistical interaction term between a host genetic variable and a microbiome variable against a drug tolerability or adverse-event outcome.

| **Drug class** | **Host-genetic determinant of response or toxicity** | **Microbial determinant of GI toxicity or drug handling** | **Records: host genetics** | **Records: microbial** | **Records: both together** | **Interaction modelled against a tolerability outcome?** |
| --- | --- | --- | --- | --- | --- | --- |
| GLP-1 receptor agonists | GLP1R and GIPR variants associated with nausea and vomiting in a genome-wide study | Microbiome regulates endogenous GLP-1 secretion and is remodelled by therapy; no human study links composition to GI adverse events | See §2 | See §3 | 9 | No — the gap this review identifies |
| Irinotecan | UGT1A1 genotype is an established determinant of irinotecan toxicity | Gut bacterial β-glucuronidase reactivates the SN-38 metabolite in the gut lumen and is linked to delayed diarrhoea | 493 | 122 | 11 | No — the eleven records retrieved were herbal or dietary interventions, a study protocol, a biomarker report and rodent mechanistic work |
| Metformin | Transporter variants are studied as determinants of response | Metformin is itself a causally active, microbiome-altering drug and a major confounder in this literature | — | 104 | — | No — the closest study used blood mRNA rather than germline genotype and fitted no interaction term |
| Levodopa | No host genetic modifier of the microbial pathway identified | A two-organism bacterial pathway, with Enterococcus faecalis tyrosine decarboxylase, metabolises levodopa before absorption | — | — | — | No — the microbial mechanism is characterised but has not been joined to host genotype |
| Digoxin | No host genetic modifier identified | Strain-level variation in Eggerthella lenta determines cardiac glycoside inactivation | — | — | — | No |
| Chemotherapy-induced mucositis | Not established in the records retrieved | A substantial literature relates microbiota composition to mucositis risk and severity | — | 86 | 4 | No — the four records retrieved concerned oral and peri-implant disease and were not relevant |
| Immune checkpoint inhibitor colitis | Not established in the records retrieved | A large literature associates gut microbiota composition with immune-related colitis | — | 373 | 1 | No — a single record retrieved, which did not fit an interaction model |

Interpretation. The pattern this review reports for GLP-1 receptor agonists is not peculiar to that drug class. Irinotecan is the clearest case: host pharmacogenetics and a microbial mechanism are each established in large, independent literatures, yet the two appear together in only eleven records and in none of them as an interaction against a toxicity outcome. The determinants are studied in parallel across pharmacology, not only here.

**Supplementary Table S4. Design and analysis considerations for a study powered to test a host-genotype-by-microbiome interaction on GLP-1 receptor agonist gastrointestinal tolerability.**

Each consideration below follows from evidence cited in the main text, identified in the final column. The table sets out what such a study would require; it is not a protocol, and the quantities given are illustrative rather than prescriptive.

| **Domain** | **Consideration** | **Basis** |
| --- | --- | --- |
| Outcome definition | Tolerability must be a prespecified primary outcome, not an adverse-event tally collected incidentally. Symptom type, severity, timing relative to each dose step, and treatment discontinuation should be distinguished, since symptom incidence and tolerability-driven withdrawal behave as different endpoints within the same trial. | Two endpoints in the same head-to-head trial diverged: near-identical symptom rates with a fourfold difference in discontinuation (§1, §5) |
| Symptom phenotyping | A validated instrument administered prospectively and repeatedly, rather than retrospective recall. Established options include the Gastrointestinal Symptom Rating Scale and, for treatment-emergent symptoms, PRO-CTCAE. Electronic diaries with event-triggered entry around dose escalation capture timing that fixed-visit assessment misses. | Self-reported outcomes in the largest pharmacogenomic study showed measurable reporting bias against record-derived estimates (§2.1) |
| Microbiome sampling | Repeat sampling is required, not a single baseline specimen. Approximately five samples per participant approaches the point beyond which additional sampling yields little further accuracy. Sampling should bracket initiation and each dose escalation, since that is when symptoms occur. | Daily profiling showed within-person variation exceeding between-person variation for most genera, with the accuracy–effort optimum at about five samples (§4.2) |
| Microbiome feature choice | Pathway- or function-level features rather than single-taxon abundance. This materially lowers the required sample size and is more robust to the compositional instability that inflates it. | Single-timepoint taxon-level testing implies 10⁴–10⁵ participants; repeat-sampled pathway-level features move the estimate toward 5,000–10,000 (§4.2) |
| Sample size | Power for the interaction, not for either main effect. Detecting a multiplicative interaction requires at least four times the sample needed for a main effect of the same magnitude, and imprecision in the interacting exposure inflates that requirement sharply. | Classical interaction power derivation, and simulation showing the requirement falling from 150,989 to below 5,000 as reliability improves (§4.2) |
| Confounding by weight loss | Weight change is on the causal path and must be separated from direct drug effects. Options include weight-matched comparison groups and mediation analysis treating weight change as a mediator rather than a covariate. | A reported microbiome shift tracked the degree of weight loss itself in a study with no weight-matched control (§3.2) |
| Confounding by concomitant metformin | A GLP-1 RA monotherapy arm is necessary. Metformin is itself microbiome-altering, and in head-to-head comparison produced a larger shift in a key taxon than the GLP-1 RA did, so any arm carrying metformin throughout cannot isolate the drug of interest. | Transfer of metformin-altered microbiota improves glucose tolerance in germ-free mice; a trial whose active arm carried metformin attributed its taxa shifts to both regimens (§3.2) |
| Confounding by diet and titration | Dietary intake and titration schedule should be recorded and modelled. Titration speed produced a larger tolerability difference than any between-drug or between-genotype comparison reported in this literature, so an unmeasured titration difference can dominate a genetic signal. | A flexible 16-week escalation reduced GI-driven withdrawal from 19% to 2% with equivalent final HbA1c (§5) |
| Statistical specification | The interaction term must be specified in advance, with multiple-testing correction appropriate to the number of microbiome features tested, and methods suited to sparse, zero-inflated and compositional abundance data. Ancestry should be modelled, given the European-ancestry skew of existing genetic evidence. | The discovery cohort of the principal genetic evidence was 78.3% European ancestry (§2.1) |
| Causal interpretation | The design should state in advance whether microbiome features are treated as causes, consequences or correlates of tolerability. The drug–microbiome relationship is bidirectional, so post-treatment composition cannot be interpreted as a baseline predictor. | Therapy remodels the microbiome, which also regulates endogenous GLP-1 secretion (§3.1, §3.2) |

Instrument references are given by name rather than by citation; specific validation references should be added if this table is retained at acceptance.
